# Supplementary material for: rDNA Copy Number Variation and Methylation in Human and Mouse Sperm
Source: Int J Mol Sci. 2025 Apr 28;26(9):4197. doi: 10.3390/ijms26094197 (PMC12071970; doi:10.3390/ijms26094197)
Supplement: Supplementary file 1 [file ijms-26-04197-s001.zip › ijms-3596465-supplementary.pdf]

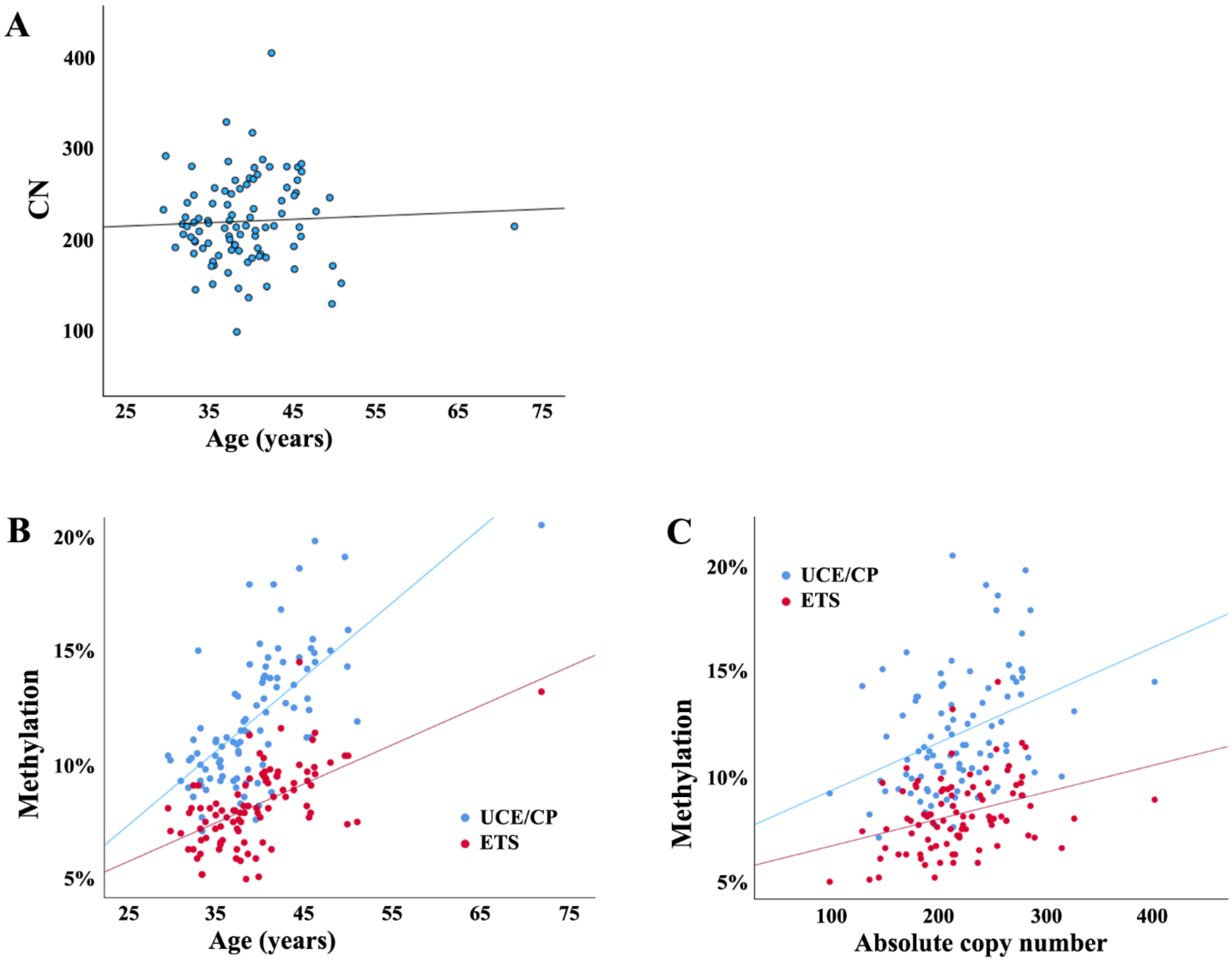

Figure S1. Absolute CN and mean methylation of the rDNA TU in human sperm samples. (A) Absolute CN of rDNA TU does not change with age in humans. Blue dots represent 94 sperm samples of males with normal semen parameters. There is no significant correlation between CN and age. (B, C) Mean methylation of the rDNA TU increases with age (B) and absolute CN (C). Blue and red dots represent mean methylation (Y axis) of the UCE/CP and the ETS region, respectively, in individual males. Both regions are positively correlated with age (UCE/CP $\rho = 0.68$, $P < 0.0001$, ETS $\rho = 0.60$, $P < 0.0001$) and absolute CN (UCE/CP $\rho = 0.38$; $P < 0.0001$ and ETS $\rho = 0.34$, $P < 0.0001$).

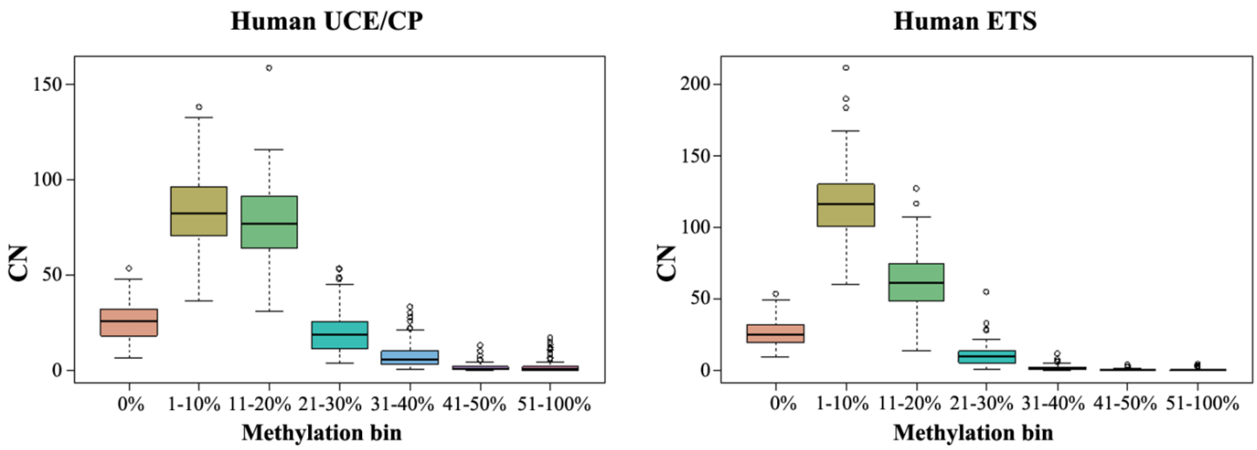

Figure S2. Number and methylation distribution of rDNA TU in human sperm samples (N = 94). Box plots showing the CN (on the Y axis) within a given methylation range in the UCE/CP and ETS region, respectively. Methylation bins representing 0%; 1-10%, 11-20%, 21-30%, 31-40%, 41-50%, and 51-100% are indicated on the X axis. The median is presented by a horizontal line. The bottom of the box indicates the 25th and the top the 75th percentile. Outliers are indicated by open circles.

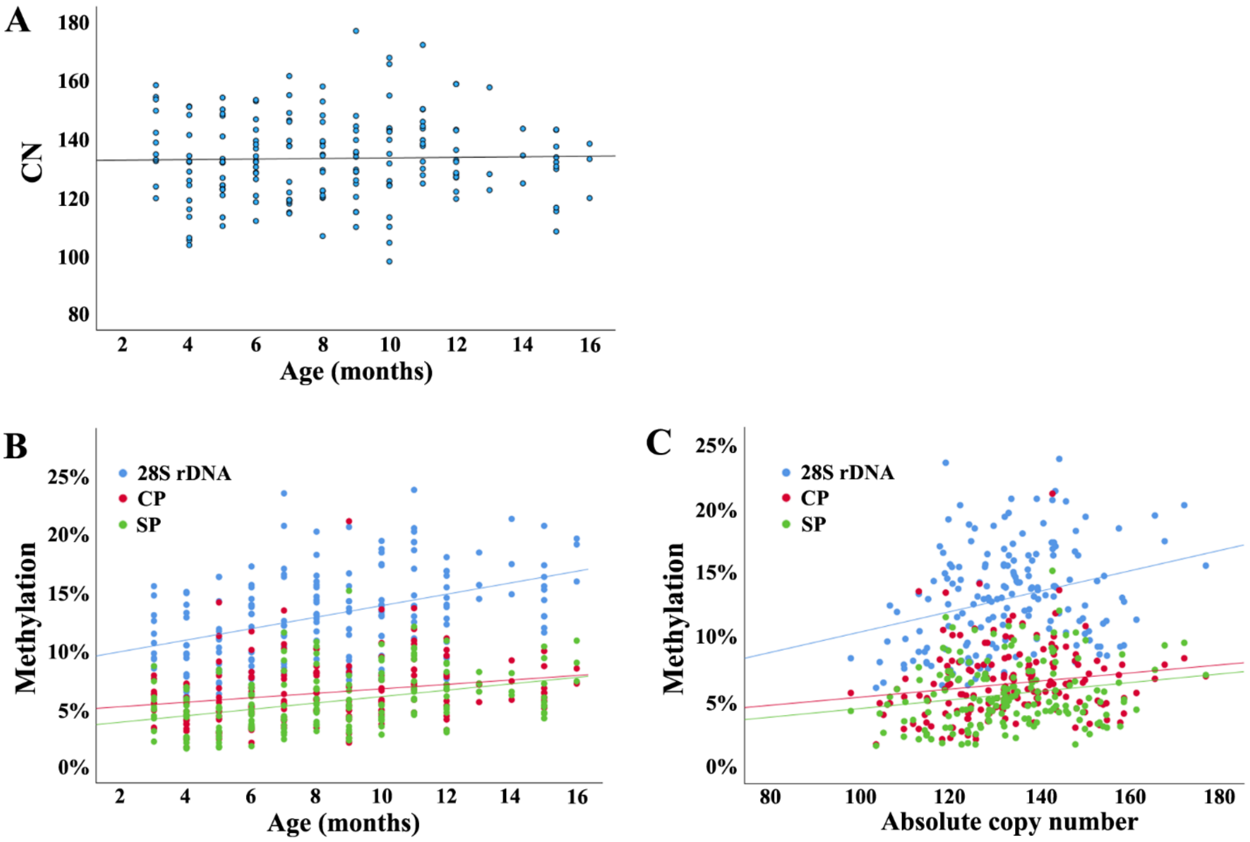

Figure S3. Absolute CN and mean methylation of the rDNA TU in mouse sperm samples (A) Absolute CN of rDNA TU does not change with age in mice. Blue dots represent 173 sperm samples from 3-16 months-old mice. There is no significant correlation between CN and age. (B, C) Mean methylation of the rDNA TU increases with age (B) and absolute CN (C). Red dots represent mean methylation (Y axis) of the core promoter (CP), green dots of the spacer promoter (SP), and blue dots of the 28S rDNA region. Methylation of all three regions is positively correlated with age (CP ρ = 0.31; $P < 0.0001$; SP ρ = 0.39; $P < 0.0001$; 28S ρ = 0.43, $P < 0.0001$) and absolute CN (CP ρ = 0.22; $P = 0.003$; SP ρ = 0.18; $P = 0.02$; 28S ρ = 0.29, $P < 0.0001$).

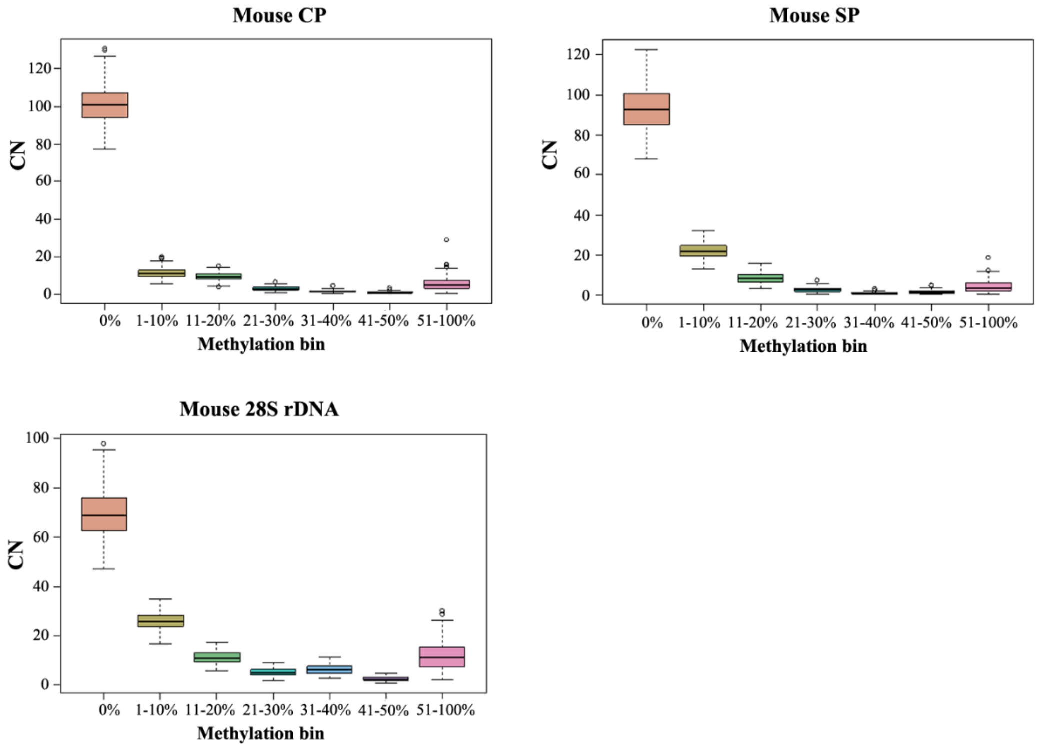

Figure S4. Number and methylation distribution of rDNA TU in mouse sperm samples (N = 173). Box plots showing the CN (on the Y axis) within a given methylation range in core promoter (CP), the spacer promoter (SP), and the 28S rDNA regions. Methylation bins representing 0%; 1-10%, 11-20%, 21-30%, 31-40%, 41-50%, and 51-100% are indicated on the X axis. The median is presented by a horizontal line. The bottom of the box indicates the 25th and the top the 75th percentile. Outliers are indicated by open circles. The majority of rDNA is completely unmethylated in all three regions. There are relatively few hypermethylated (> 10%) copies.

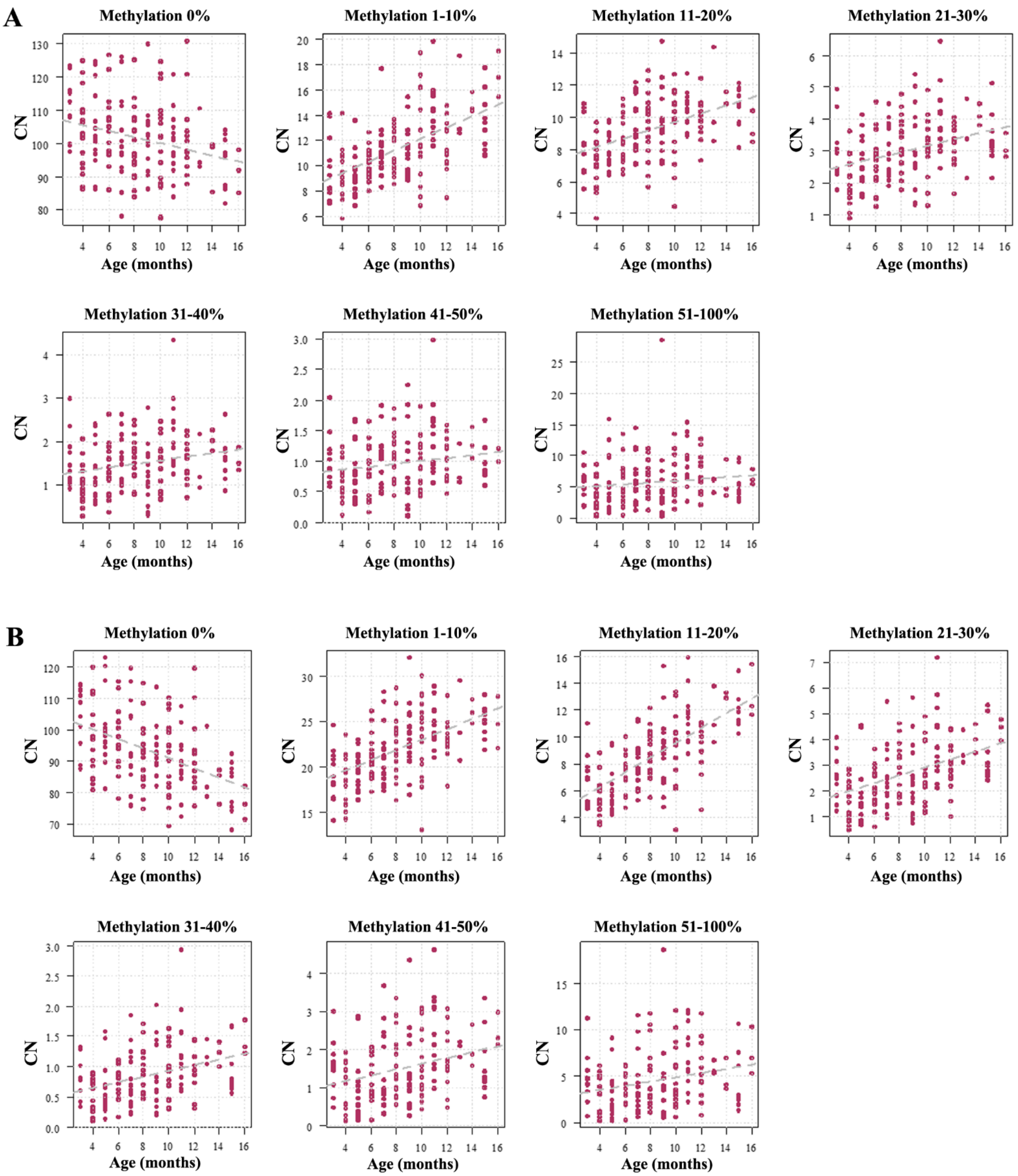
A
Methylation 0%
Methylation 1-10%
Methylation 11-20%
Methylation 21-30%
Methylation 31-40%
Methylation 41-50%
Methylation 51-100%
B
Methylation 0%
Methylation 1-10%
Methylation 11-20%
Methylation 21-30%
Methylation 31-40%
Methylation 41-50%
Methylation 51-100%
CN
Age (months)

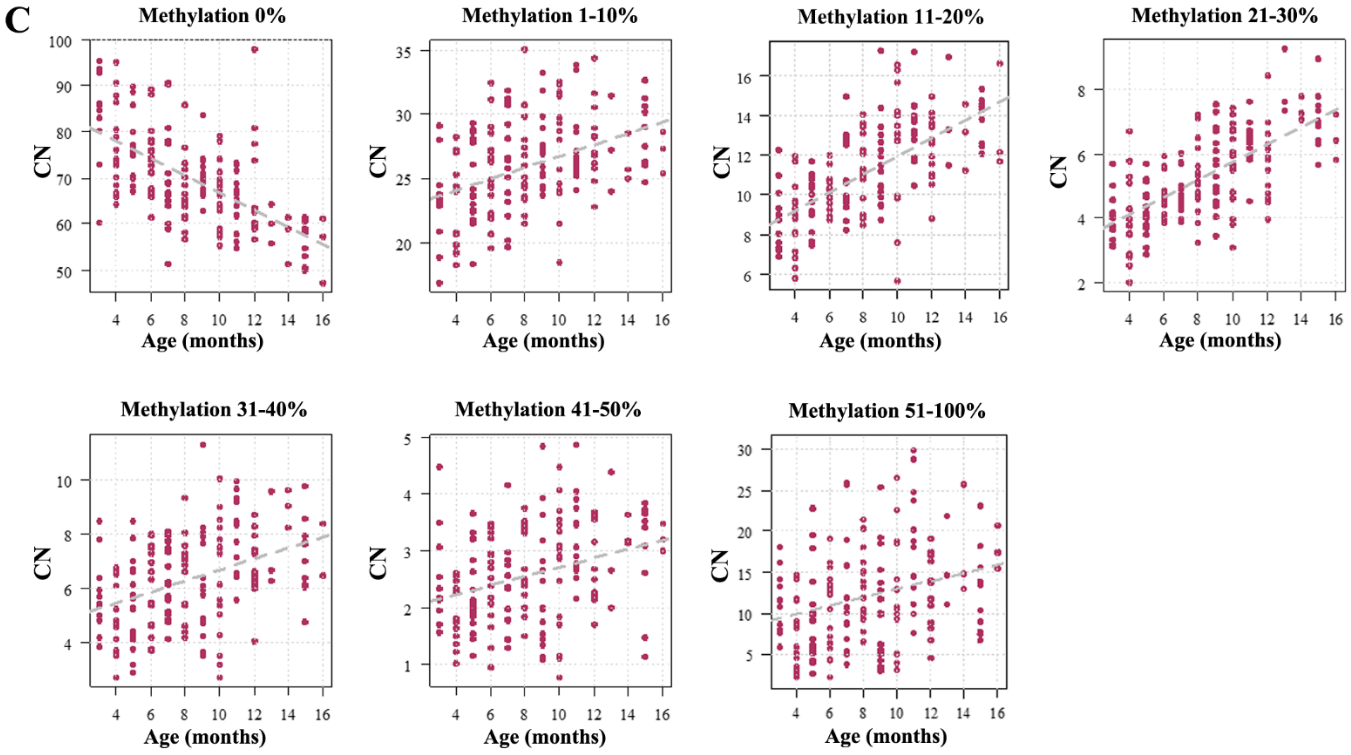

Figure S5. Age-related methylation changes of the rDNA core promoter (A), spacer promoter (B), and 28S rDNA (C). The Y axis shows the number of rDNA TU within a given methylation bin. Each dot represents an individual mouse sperm sample (N = 173). For the CP, the SP and the 28S rDNA, the number of completely unmethylated (0%) TU decreases with age, whereas the CN in the 1-10%, 11-20%, 21-30%, 31-40%, 41-50%, and 51-100% methylation bins increased. For the CP (A), the Spearman correlations are: 0% (ρ = -0.28, P = 0.0002), 1-10% (ρ = 0.57, P < 0.0001), 11-20% (ρ = 0.51, P < 0.0001), 21-30% (ρ = 0.57, P < 0.0001), 31-40% (ρ = 0.27, P = 0.0004), 41-50% (ρ = 0.21, P = 0.004), and 51-100% (ρ = 0.18, P = 0.018). For the SP (B): 0% (ρ = -0.44, P < 0.0001), 1-10% (ρ = 0.58, P < 0.0001), 11-20% (ρ = 0.71; P < 0.0001), 21-30% (ρ = 0.44, P < 0.0001), 31-40% (ρ = 0.35, P < 0.0001), 41-50% (ρ = 0.31, P < 0.0001), and 51-100% (ρ = 0.26, P = 0.0005). For 28S rDNA: 0% (ρ = -0.63, P < 0.0001), 1-10% (ρ = 0.43, P < 0.0001), 11-20% (ρ = 0.67, P < 0.0001), 21-30% (ρ = 0.68, P < 0.0001), 31-40% (CP ρ = 0.27, P = 0.0004; SP ρ = 0.35, P < 0.0001; 28S rDNA ρ = 0.40, P < 0.0001), 41-50% (ρ = 0.34, P < 0.0001), and 51-100% (ρ = 0.29, P = 0.0001).

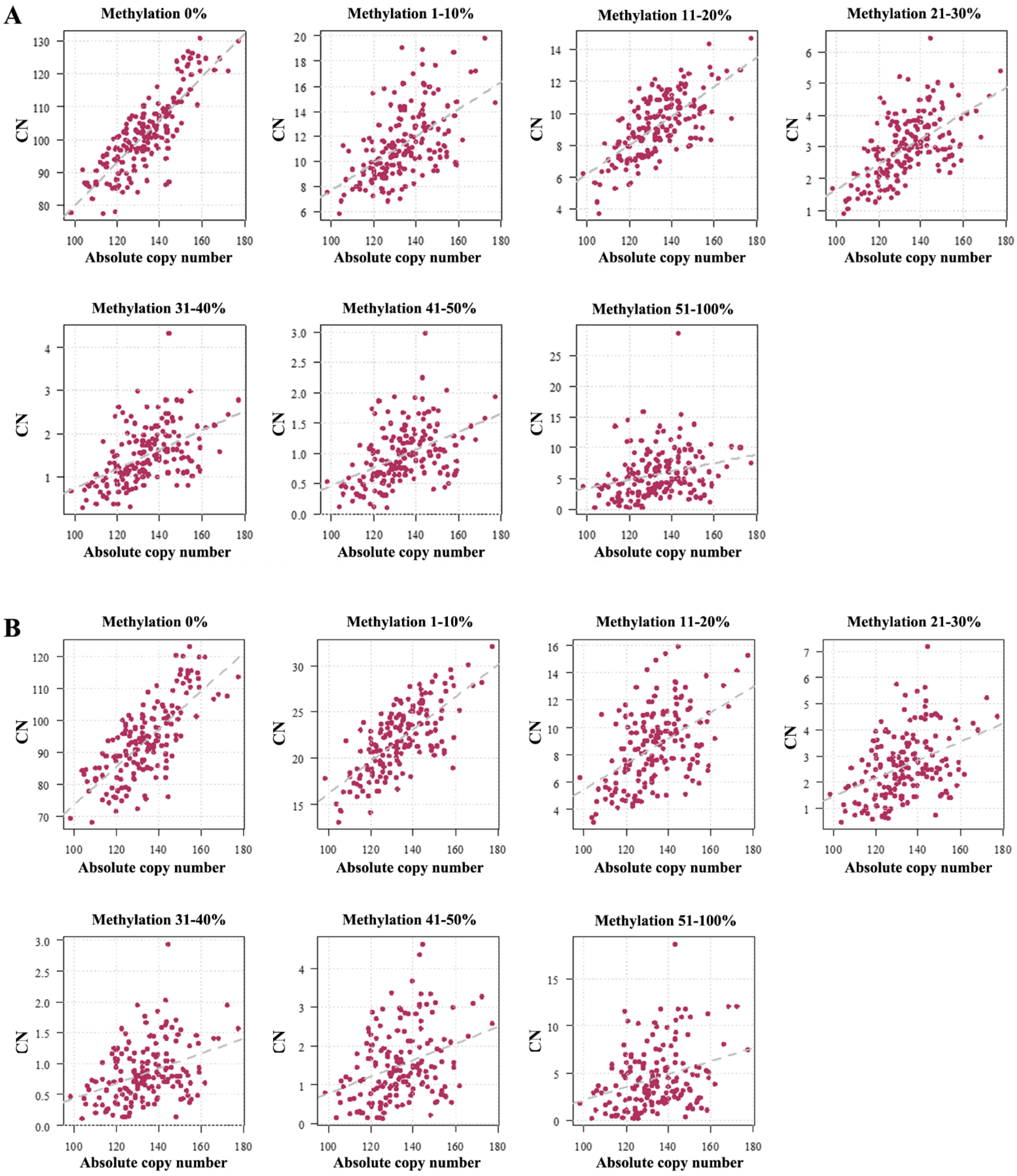
A
Methylation 0%
Methylation 1-10%
Methylation 11-20%
Methylation 21-30%
Methylation 31-40%
Methylation 41-50%
Methylation 51-100%
CN
Absolute copy number
B
Methylation 0%
Methylation 1-10%
Methylation 11-20%
Methylation 21-30%
Methylation 31-40%
Methylation 41-50%
Methylation 51-100%
CN
Absolute copy number

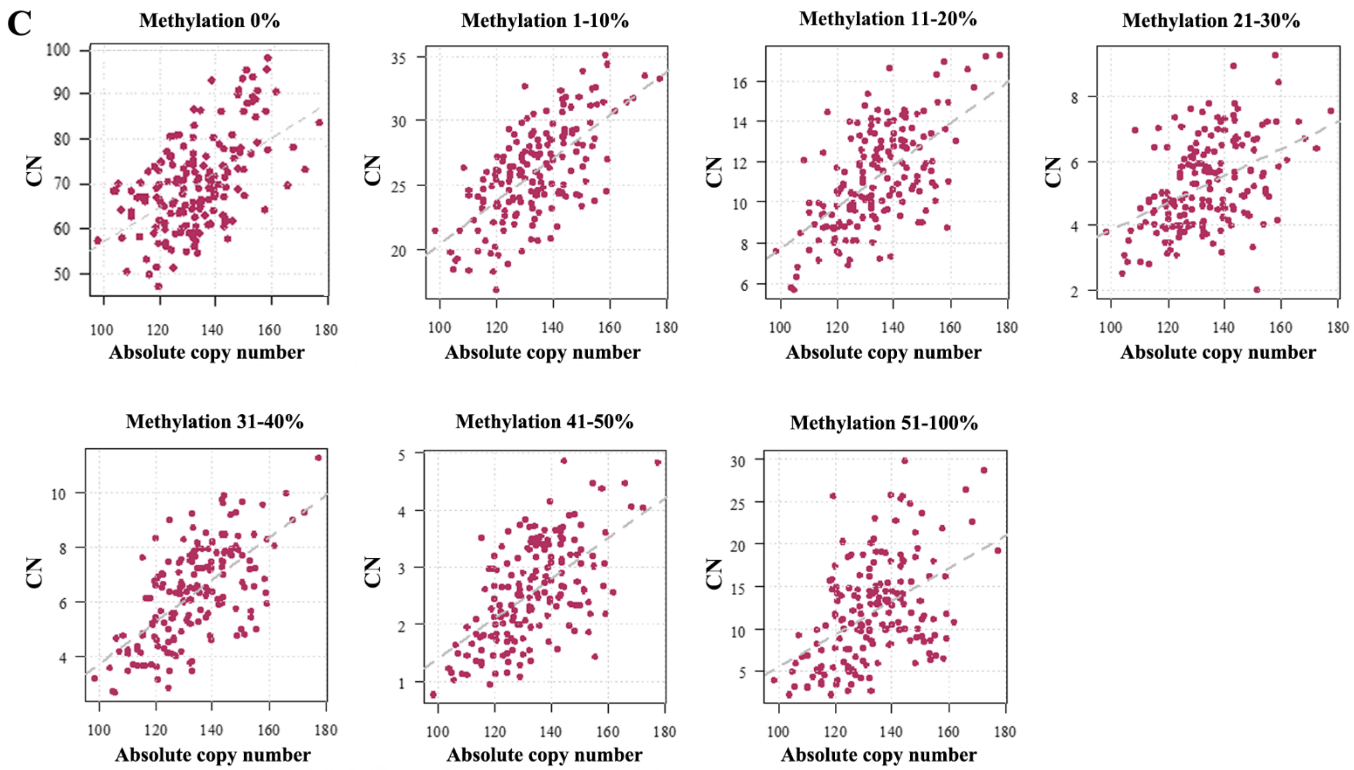

Figure S6. Core promoter (A), spacer promoter (B), and 28S rDNA (C) methylation increases with absolute CN. Each dot represents an individual mouse sperm sample (N = 173). The X axis shows the absolute number of rDNA TU copies. The Y axis presents the number of rDNA TU within a given methylation bin (0%, 1-10%, 11-20%, 21-30%, 31-40%, 41-50%, and 51-100%). The CN of all three regions in all bins is increasing with absolute CN. For the CP (A), the Spearman correlations are: 0% ($\rho = 0.80$, $P < 0.0001$), 1-10% ($\rho = 0.57$, $P < 0.0001$), 11-20% ($\rho = 0.65$), 21-30% ($\rho = 0.59$, $P < 0.0001$), 31-40% (CP $\rho = 0.53$, $P = 0.0004$), 41-50% ($\rho = 0.50$, $P < 0.0001$), and 51-100% ($\rho = 0.32$, $P < 0.0001$). For the SP (B): 0% ($\rho = 0.70$, $P < 0.0001$), 1-10% ($\rho = 0.67$, $P < 0.0001$), 11-20% ($\rho = 0.48$; $P < 0.0001$), 21-30% ($\rho = 0.39$, $P < 0.0001$), 31-40% ($\rho = 0.37$, $P < 0.0001$), 41-50% ($\rho = 0.33$, $P < 0.0001$), and 51-100% ($\rho = 0.29$, $P = 0.0001$). For 28S rDNA (C): 0% ($\rho = 0.49$, $P < 0.0001$), 1-10% (CP $\rho = 0.57$, $P < 0.0001$; SP $\rho = 0.67$, $P < 0.0001$; 28S $\rho = 0.62$, $P < 0.0001$), 11-20% $\rho = 0.57$, $P < 0.0001$), 21-30% ($\rho = 0.43$, $P < 0.0001$), 31-40% ($\rho = 0.61$, $P < 0.0001$), 41-50% ($\rho = 0.55$, $P < 0.0001$), and 51-100% ($\rho = 0.44$, $P < 0.0001$).

Table S1. Primers for droplet digital PCR of rDNA

Species	Assay	Primer	Sequence (5'-3')[a]
Human	28S rDNA	Forward	5′-AACGTGAGCTGGGTTTAG-3′
		Reverse	5′-CTCGTACTGAGCAGGATTAC-3′
		Probe	5′-/5HEX/TGGCAACAA/ZEN/CACATCATCAGT/3IABkFQ/-3′
	TBP	Forward	5′-GATATGAGACTGTGGGTAAGT-3′
		Reverse	5′-GATCCTTTGAACACCCTAATG-3′
		Probe	5′-/56-FAM/ACAGAGATC/ZEN/ACTGCAGTTGC/3IABkFQ/-3′
Mouse	28S rDNA	Forward	5′-AACGTGAGCTGGGTTTAG-3′
		Reverse	5′-CTCGTACTGAGCAGGATTAC-3′
		Probe	/5HEX/TGGCAACAA/ZEN/CACATCATCAGT/3IABkFQ/
	Gapdh	Forward	5′-AGCCTTTACTACAGAACATCTCAC-3′
		Reverse	5′-TCTTTCCTCTCCCTTCCCTTTA-3′
		Probe	/56-FAM/ACTCCAACA/ZEN/AATGCTTGCTGACGC/3IABkFQ/

Table S2. Primers for deep bisulfite sequencing of the rDNA TU

Essay	Primer	Sequence (5'-3')[a]	Amplicon length	Variant	Annealing Temp. (^oC)	No. of CpGs
Human rDNA CP and UCE	Forward	TATTYGGAGGTTTAATTTTTTTAG	239 bp	A/G*	56°C	25
	Reverse	TATATCCTAAAATTAACCAAAAAACCCC				
Human rDNA ETS	Forward	GGAGTTAGYGGGGTGGGGTTGT	271 bp	A*/G	56°C	38
	Reverse	ACTAAAAAAATTAAACCTCC				
Mouse rDNA CP	Forward	TTGGGGAGGTGGTTTAAAAATGA	189 bp	-	60°C	10
	Reverse	CCTCCAAAAACCCTCTCTAT				
Mouse rDNA SP	Forward	GGAGAAGTGGTGGGTGGG	219 bp	-	60°C	17
	Reverse	CTCCTATATCACCAACCTAAAAAACCT				
Mouse 28S rDNA	Forward	GGTTTTAAGTAGGAGGTGTTAGAAAAG	292 bp	-	60°C	13
	Reverse	CAACCAAACACATACACCAAATATCT				

* indicates the major allele
